# Fatigue in Emergency Services Operations: Assessment of the Optimal Objective and Subjective Measures Using a Simulated Wildfire Deployment

**DOI:** 10.3390/ijerph13020171

**Published:** 2016-01-29

**Authors:** Sally A. Ferguson, Bradley P. Smith, Matthew Browne, Matthew J. Rockloff

**Affiliations:** 1 Central Queensland University, Appleton Institute, Adelaide 5034, Australia; b.p.smith@cqu.edu.au; 2 School of Human Health and Social Sciences, Central Queensland University, North Rockhampton QLD 4701, Australia; m.browne@cqu.edu.au (M.B.); m.rockloff@cqu.edu.au (M.J.R.)

**Keywords:** fatigue, multi-stressor, self-assessment, subjective, objective, fire fighters

## Abstract

Under controlled laboratory conditions, neurobehavioral assays such as the Psychomotor Vigilance Task (PVT) are sensitive to increasing levels of fatigue, and in general, tend to correlate with subjective ratings. However, laboratory studies specifically curtail physical activity, potentially limiting the applicability of such findings to field settings that involve physical work. In addition, laboratory studies typically involve healthy young male participants that are not always representative of a typical working population. In order to determine whether these findings extend to field-like conditions, we put 88 Australian volunteer firefighters through a multi-day firefighting simulation. Participants were required to perform real-world physical and cognitive tasks under conditions of elevated temperature and moderate sleep restriction. We aimed to examine changes in fatigue in an effort to determine the optimum objective and subjective measures. Objective and subjective tests were sensitive to fatigue outside laboratory conditions. The PVT was the most sensitive assay of objective fatigue, with the Samn-Perelli fatigue scale the most sensitive of the subjective measures. The Samn-Perilli fatigue scale correlated best with PVT performance, but explained a small amount of variance. Although the Samn-Perelli scale can be easily administered in the field, the wide range of individual variance limits its efficacy as a once-off assessment tool. Rather, fatigue measures should be applied as a component of a broader fatigue risk management system. Findings provide firefighting agencies, and other occupations involving physical work, guidance as to the most sensitive and specific measures for assessing fatigue in their personnel.

## 1. Introduction

Fatigue arises as a result of task related factors (e.g., task demand), inadequate sleep, circadian misalignment and extended time awake [1,2]. Management of fatigue-related risk in operations that use long hours and/or night work requires multiple levels of control [3,4,5]. While almost all fatigue mitigation strategies involve organizational controls such as setting maximum limits on work hours or minimum rest break requirements, others may incorporate controls at the level of the individual. In order to identify instances of increased risk, both objective fatigue monitoring and subjective fatigue reporting can be used [5]. These methods are based on the premise that in situations where sleep is inadequate, cognitive performance decreases and subjectively, people report they are tired or fatigued [5,6,7]. Assessment of fatigue levels to identify increased fatigue-related risk therefore requires measures that are sensitive to changes across time and under a range of conditions.

Neurobehavioral assays such as the Psychomotor Vigilance Task have consistently shown to be sensitive to increasing levels of fatigue, demonstrated by numerous laboratory studies [8,9]. In addition, objective performance correlates reasonably well with subjective ratings of fatigue [10]. However, laboratory studies specifically curtail physical activity, potentially limiting the applicability of findings to field settings that involve physical work. Akerstedt and colleagues [11] reported that reductions in ratings of sleepiness are dependent on context, including “free activity”. Thus, if physical activity masks subjective feelings of sleepiness or fatigue, self-reports may not be appropriate in occupational settings that involve physical work. Assessment of measures of fatigue in the presence of physical activity and personal protective equipment (e.g., helmets, gloves, jackets, boots) is an important current gap in the literature.

The current study examined changes in fatigue measured using both objective and subjective tests in a simulated wildfire deployment. The environmental and occupational stressors experienced during wildfire suppression operations are associated with elevated likelihood of fatigue—extended work shifts, night work, inadequate sleep opportunity and inadequate sleeping environments, with implications for health and safety of personnel [12,13]. The simulation involved real-world physical tasks that are performed routinely by Australian tanker-based firefighters, under conditions of elevated ambient temperature and moderate sleep restriction. The aims were to determine the most sensitive objective and subjective measures to fatiguing conditions, in a population of active firefighters. Findings will provide firefighting agencies, and other occupations involving physical work, guidance as to the most sensitive and specific measures for assessing fatigue in their personnel.

## 2. Experimental Section

### 2.1. Participants

A total of 88 healthy active volunteer rural fire fighters (Males, *N* *=* 77, Females; *N* *=* 11) took part in the study. The participants had an overall mean (±SD) age of 38.42 ± 14.42, and an average body mass index (BMI) of 27.8 kg/m² ± 4.53, reflective of Australian bush firefighters [14]. Participants, who volunteered to take part in the study, were recruited from various state and territory rural fire agencies across Australia. Exclusion criteria included current or pre-existing injury or condition preventing performance of fire ground duties, diagnosed sleep disorder, and pregnancy. Participants were randomly assigned to either the normal sleep/cool (*n* = 29), normal sleep/hot (*n* = 20), sleep restricted/cool (*n* = 26), or sleep restricted/hot (*n* = 13) condition (see Section 2.2). Participants self-selected suitable dates for testing but were not aware of the condition ahead of testing. A total of 21 study trials were conducted across three locations. The number of participants in each study trial ranged from 2 to 5. A total of 98 participants began the trials, however 10 voluntarily withdrew at various points in the trial (these participants were excluded from the analysis).

### 2.2. Procedure

**Figure 1:**
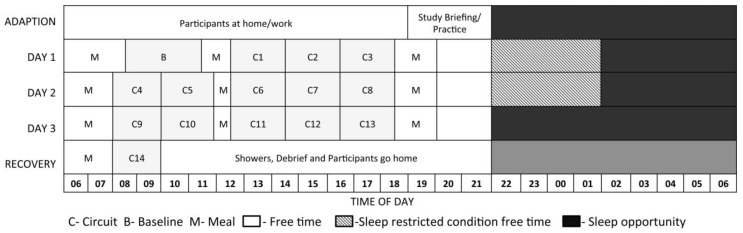
The study protocol outlining each testing circuit, scheduled meal times, free time, and sleep opportunity. Participants in the sleep restricted conditions were given an extended free time.

We developed a laboratory-based simulation of a fire-ground tour that incorporated three consecutive 12-h day shifts [15]. The study protocol spanned four days, including a study briefing, familiarisation of the tasks, and adaption to sleeping conditions (stretcher bed) on the evening prior to testing (arrival 6 pm), and a morning testing session on day four. Participants lived in a simulated environment for the duration of the study and remained inside except when using external amenities. Refer to Figure 1 for an outline of the protocol. All components of the simulation, including day and night temperatures, sleeping environment, as well as physical and cognitive test batteries were designed in conjunction with subject matter experts and using field data [15].

Participants completed 15, two-hour testing sessions over 4 days. Each session consisted of 55 min of physical work designed to mimic fire-ground tasks (see Section 2.3), followed by physiological testing lasting 20 min, and a cognitive battery lasting 20 min. Testing sessions were completed in the participants own firefighting personal protective clothing throughout the simulation. This included a two-piece jacket and trouser set made from Proban cotton fabric (Solvay, Brussels, Belgium), suspenders, boots, gloves, helmet, and goggles (amounting to ~5 kg). On completion of each session, participants had a 15–30 min break before beginning the next session. Participants were not required to wear any protective equipment during meal breaks, physiological testing, between testing sessions, or during free time. Cognitive testing sessions were completed in full personal protective equipment with the exception of gloves.

All participants were given an 8 h sleep opportunity (10:00 pm–6:00 am) on the adaptation night (night 1), and the recovery night (night 4). In the normal sleep/cool and normal sleep/hot conditions, participants were also given 8 h sleep opportunity on nights 2 and 3. However, in the sleep restricted/hot and sleep restricted/cool conditions, participants were given 4 h sleep opportunity (2 am–6 am) on nights 2 and 3. Ambient temperature for participants in the normal sleep/cool and sleep restricted/cool conditions was kept to 18–20 Degrees Celsius (C) during the day and night, For the sleep restricted/hot and sleep restricted/cool conditions, the temperature was raised to 33–35 C during the day (between 6 am–6 pm) and lowered to 20–23 C during the night (6 pm–6 am). Temperature was monitored using HOBOware Pro Software, and three ZW-003 Temperature Data Nodes (Onset Computer Corporation, Bourne, MA, USA) positioned around the room. All subjects gave their informed consent for inclusion before they participated in the study. The study was conducted in accordance with the Declaration of Helsinki, and the protocol was approved by the Ethics Committee of Central Queensland University (H12/01-016) and Deakin University Human Research Ethics Committees (210-170).

### 2.3. Physical Tasks

Participants were required to complete a series of six physical tasks designed to simulate real-world fire-fighting [16] during each 55 min circuit. During a testing circuit, each firefighter was placed on a specific work-rest schedule, and rotated through a series of 5-min tasks. Tasks included dragging a weighted tyre, raking debris, different forms of walking with a weighted hose whilst avoiding obstacles, holding a weighted hose rake in a static position, and rolling up a 25 m fire hose to operational standard (see paper by Vincent *et al.*, [17], that resulted from the outcomes of the same study for details relating to the physical tasks).

### 2.4. Cognitive Tasks

The cognitive test battery included tasks that require both lower order (e.g., reaction time) and higher order tasks (e.g., requiring executive function) to tap a range of cognitive functions used by personnel during incident response [15] and reported to be variously sensitive to fatigue in laboratory settings (see below).

Psychomotor Vigilance Task (PVT): The PVT assesses vigilance and reaction time and is sensitive to the effects of sleep loss, time-on-task and time-of-day [8,18,19]. While longer tests are commonly used in the laboratory, a validated 5-min version, run on a palm pilot, was used due to time constraints [18]. PVT variables used in this study include mean reciprocal reaction time calculated by 1/RT*1000 [6,8], and lapses: which classifies RT ≥500 milliseconds as a lapse in attention.

Memory Task: Participants were given 20 s to read an authentic emergency message on a handheld pager device (SAGRN Samsung SFA-170 Pager, Samsung). After a 5-min gap (when they were completing the PVT), participants were given 2 min to recall three aspects of the message that differed each time (either the incident number, location of incident, type of incident, and map reference). The maximum score that could be achieved for each testing session was 3 (1 point given for each aspect recalled).

Stroop Colour Word Test: Participants were required to respond to two different stimuli over two different tests- a “matching colour word”, and a “non-matching colour word” task [20,21]. Each component lasted 2 min. In the matching colour-word condition, participants were presented with a series of coloured words (e.g., red blue, yellow and green) displayed on a black computer screen. Participants were instructed to select the colour of the text/font by pressing the corresponding colour key on a colour coded computer keypad. In the non-matching colour word condition, the colour of the word and the word were different (e.g., the word red was written in green font). Participants were required to select the colour (font) of the word, and not the word itself. This second task produces a phenomenon known as the “stroop effect”, where response time is delayed in the second part of the task. That is, there is a cognitive trade-off between speed and accuracy. Percentage correct and reaction time for correct responses were used as the dependent variables for this test.

Go-No-Go: The Go-No-Go is a task that requires the participant to inhibit their responses. In this task, the participant must differentiate between responding to “go” stimuli, interspersed with “no go” (no response required) stimuli which have a lower rate of presentation frequency (see [22]). The Go No-Go task presented four shape stimuli in the center of the screen for a period of 200 ms, followed by a black screen interval of 1300 ms, three of the shape stimuli were respond or “go stimuli” and one was no response or “no-go”, stimuli in which they still had time to respond. Each test went on average for 4 mins 35 s, with 181 images shown and 63% (median of 61.88%–68.3%) of these consisting of “go” images. Participants were instructed by researchers to press the space bar as quickly as possible, with their dominant index finger, right or left, in response to the “go images”, and then refrain from pressing the space bar when the no-go images appear, Percentage (%) correct: Stimuli responded to that were “go” stimuli; and stimuli left for 1.5 ms that were “no go” stimuli. Go-No-Go was measured as percentage correct, and reaction time.

Occupational Safety Performance Assessment Test (OSPAT): The OSPAT is an unpredictable tracking task that assesses hand-eye coordination, sustained attention and reaction time, and is widely used as a fitness for duty measure in a variety of industries (see [23]). For this task, participants were instructed to keep the computer cursor positioned on the target displayed on the screen as much as possible for the duration of the test. This task lasted 60 s. A global performance measure for each test is determined by summing the “error” distance between the cursor and target and the rate at which the subject adapted to the random changes. This measure indicated how “well” the subject performed on the task. Scores typically fall between 10 and 20 with higher scores indicating better performance. The algorithm used to determine the dependent measure derived from OSPAT cannot be disclosed as it is subject to commercial confidence.

### 2.5. Subjective Measures

Prior to cognitive testing, participants were required to assess their own level of fatigue using the Samn-Perelli Fatigue Scale, where 1 = fully alert, wide awake and 7 = completely exhausted, unable to function effectively [24]. Participants were also required to rate their motivation to perform the next cognitive battery, alertness at that time point, and predicted performance on the next cognitive battery using visual analogue scales [25]. That is, they responded to the question “How motivated are you to perform well?” by placing a single stroke at the applicable point along a 100 mm line, with the anchors “not motivated at all” at the left-hand end and “very motivated” at the right-hand end; “How alert do you feel?” with anchors “not alert at all” on the left and “very alert” on the right; and “How well do you think you will perform?” with anchors “very poorly” on the left, and “very well” on the right.

## 3. Results

### 3.1. Objective Measures

To assess the objective measure over the 15 sessions of the study that best reproduced the true fatigue of the subjects, we made a fundamental assumption. There were several days of testing, and several sessions within each day (see Figure 1). We used the very last session prior to a recovery period (Circuit 13), and assumed that this was a period of maximum fatigue for most subjects. Conforming to this assumption, mean performance, as a composite of all standardised objective and subjective measures, was worst on this day. Baseline (Circuit B) and Recovery (Circuit 14) sessions had the highest mean performance, and were used as comparison sessions.

Our data-analytic approach, therefore, became to find the best measure that showed maximum responsiveness to demonstrating fatigue in this test condition, relative to the comparison conditions. We went about this analysis in two ways: In the first step, we analysed which measure showed maximum responsiveness to the change in performance from the first “baseline” measure where participants were not fatigued, to the final session where they experienced the most fatigue. Next, we repeated this analysis to determine which measures showed the maximum responsiveness from this test condition of maximum fatigue to the recovery period on the following day.

As a proportion, Go-No-Go Percentage Correct was arcsin transformed to yield an approximately normal response. All other measures of objective fatigue were approximately normal, except for some moderately extreme values that had the potential to bias the analyses. A lower-bound threshold for OSPAT was set at −8 (below which scores were set to −8), whilst upper-bound thresholds (above which, scores were set to the threshold), Stroop Reaction Time (1.5) and PVT Lapses (2). Thresholding affected less than 1% of OSPAT, Stroop Reaction Time, and PVT Lapses scores. These transformations had the effect of making the test of differences more conservative by stabilizing the variance of the measures.

In the study, subjects were assigned to conditions of normal sleep/cool, sleep restricted/cool, normal sleep/hot, or sleep restricted/hot. Although all subjects were subject to fatigue, our assumption is that the sleep restricted/hot conditions produced a particular stress on subjects, and the other conditions should have a variable (and expected lessor) impact on the experience of fatigue. Therefore, we also included in our analysis a between-subjects consideration of how condition assignment affected these fatigue change scores. All change-score residuals conformed to the Shapiro-Wilk test of normality, except for Stroop reaction time (W = 0.965, *p* = 0.016). Inspection of the residual distribution confirmed that it was symmetrical and mildly platykurtic. Therefore, this violation of assumptions was not treated as a concern.

Table 1 shows the result of ANOVA models applied to each change measure, with condition (treated as a single 4 level factor) as the independent variable. Notably, we excluded an intercept term in the model to assess the significance of the deviation of each score from 0 (*i.e.*, no change from baseline). Panel A in Table 1 shows the results for changes from the baseline measure to the test condition with maximum fatigue. The PVT Mean Reaction Time shows the strong performance amongst the measures by demonstrating a strong deviation from the null hypothesis of no change ($r^{2}=0.41$). Go-No-Go reaction time deviated slightly more from baseline ($r^{2}=0.48$). However, the PVT Mean Reaction Time shows stronger responsiveness than the Go-No-Go to the sleep restricted/hot manipulations compared to the other less stressed conditions.

Table 1 Panel B shows the changes from the test condition with maximum fatigue to the recovery period the following day. The PVT Mean Reaction Time shows the best performance by demonstrating maximum change ($r^{2}=0.40$), as well as the greatest absolute change for the sleep restricted/hot condition of subjects that experienced the greatest stress. In this case, Go-No-Go reaction time was markedly less sensitive overall ($r^{2}=0.18$).

Table 1ANOVAs predicting changes in outcome scores for objective measures. Models for each outcome (7) were run separately for changes from baseline to test (**Panel A**) and test to recovery (**Panel B**) **^**.

**Panel A:** Changes baseline to test.

Condition	Model: OSPAT	Model: Memory	Model: PVT Mean Reaction Time	Model: Stroop % Correct	Model: Stroop Reaction Time	Model: Go-No-Go % Correct	Model: Go-No-Go Reaction Time
Sleep restricted/cool	0.40	0.09	−0.43 ***	−0.01	−0.02	−0.01	0.05 ***
	(0.27)	(0.22)	(0.09)	(0.03)	(0.03)	(0.03)	(0.01)
Sleep restricted/hot	−0.24	−0.46	−0.63 ***	−0.01	0.03	−0.07	0.06 ***
	(0.38)	(0.31)	(0.13)	(0.04)	(0.04)	(0.04)	(0.01)
Normal sleep/cool	0.03	0.50 *	−0.26 **	−0.05	−0.06 *	−0.00	0.03 **
	(0.25)	(0.21)	(0.08)	(0.03)	(0.03)	(0.02)	(0.01)
Normal sleep/hot	0.08	0.17	0.08	−0.03	−0.10 **	0.00	0.04 ***
	(0.31)	(0.25)	(0.10)	(0.03)	(0.03)	(0.03)	(0.01)
R^2^	0.03	0.09	0.41	0.06	0.17	0.04	0.48
Adj. R^2^	−0.01	0.05	0.38	0.01	0.13	−0.00	0.45
Num. obs.	88	88	88	88	88	88	88
*F*(4,94)	0.68	2.12	14.60 **	1.27	4.17 **	.90	19.28 ***

*** *p* < 0.001, ** *p* < 0.01, * *p* < 0.05.

**Panel B:** Changes test to recovery.

Condition	Model: OSPAT	Model: Memory	Model: PVT Mean Reaction Time	Model: Stroop % Correct	Model: Stroop Reaction Time	Model: Go-No-Go % Correct	Model: Go-No-Go Reaction Time
Sleep restricted/cool	0.25	−0.31	0.35 ***	−0.00	−0.07 **	0.06 **	−0.02 *
	(0.28)	(0.19)	(0.07)	(0.02)	(0.02)	(0.02)	(0.01)
Sleep restricted/hot	0.48	0.38	0.54 ***	0.03	−0.11 **	0.09 **	−0.04 ***
	(0.39)	(0.27)	(0.10)	(0.03)	(0.03)	(0.03)	(0.01)
Normal sleep/cool	0.28	−0.12	0.13	0.02	−0.03	0.06 ***	−0.00
	(0.26)	(0.18)	(0.07)	(0.02)	(0.02)	(0.02)	(0.01)
Normal sleep/hot	−0.17	−0.24	0.14	0.06 **	−0.04	0.05 *	−0.02 *
	(0.32)	(0.22)	(0.08)	(0.02)	(0.03)	(0.02)	(0.01)
R^2^	0.04	0.07	0.40	0.10	0.21	0.31	0.22
Adj. R^2^	−0.00	0.02	0.38	0.06	0.18	0.28	0.18
Num. obs.	88	88	88	88	88	88	88
*F*(4,84)	0.94	1.52	14.21 ***	2.44	5.69 ***	9.61 ***	5.89 ***

*** *p* < 0.001, ** *p* < 0.01, * *p* < 0.05. **^** Table values are standardized beta-weights with standard errors in parentheses. No intercept term was included in these models. Therefore, significance effects are deviations from 0 and comparison conditions.

### 3.2. Subjective Measures

The analysis outlined above was repeated for the 4 subjective measures: Fatigue (Samn-Perelli Fatigue Scale), Alertness, Motivation, and Pre-Performance. The purpose of this analysis, as outlined in Table 2, was to find the subjective measure that showed the greatest responsiveness to our experimental session with the highest induced fatigue by comparing it with the baseline (see Table 2, Panel A) and the recovery session (see Table 2, Panel B). Moreover, as with the objective measures, we assumed that the best measure would show greatest responsiveness, in terms of a change score, for the sleep restricted/hot condition. This analysis showed that all measures of subjective fatigue performed reasonably well in detecting deviations from baseline to test. However, the Samn-Perelli Fatigue Scale measure yielded the greatest variance in both the change in baseline to test ($r^{2}=0.60$) and the change in test to the recovery period ($r^{2}=0.70$). Similarly, all measures of subjective fatigue showed the relative best performance in being sensitive to changes for those participants in the most stressed sleep restricted/hot condition. However, the Samn-Perelli Fatigue Scale subjective measure was again superior (β = 0.90) in terms of sensitivity to this condition relative to other conditions and other measures.

Table 2ANOVAs predicting changes in standardised outcome scores for subjective measures. Models for each outcome (7) were run separately for changes from baseline to test (**Panel A**) and test to recovery (**Panel B**).

**Panel A:** Changes baseline to test.

Condition	Model: Samn-Perelli Fatigue Scale	Model: Alertness	Model: Pre-Performance	Model: Motivation
Sleep restricted/cool	1.32 ***	−14.90 ***	−10.16 *	−0.23 **
	(0.29)	(4.21)	(3.99)	(0.08)
Sleep restricted/hot	3.08 ***	−40.62 ***	−32.92 ***	−0.49 ***
	(0.41)	(5.96)	(5.64)	(0.11)
Normal sleep/cool	1.39 ***	−16.83 ***	−12.55 **	−0.30 ***
	(0.27)	(3.99)	(3.77)	(0.07)
Normal sleep/hot	1.58 ***	−19.81 ***	−24.74 ***	−0.30 ***
	(0.33)	(4.80)	(4.54)	(0.09)
R^2^	0.60	0.53	0.49	0.42
Adj. R^2^	0.58	0.51	0.47	0.39
Num. obs.	88	88	88	88
*F*(4,84)	31.32 ***	23.45 ***	20.34 ***	15.13 ***

*** *p* < 0.001, ** *p* < 0.01, * *p* < 0.05*.*

**Panel B:** Changes test to recovery.

Condition	Model: Samn-Perelli Fatigue Scale	Model: Alertness	Model: Pre-Performance	Model: Motivation
Sleep restricted/cool	−1.21 ***	12.03 **	6.93 *	0.10
	(0.24)	(4.06)	(3.46)	(0.06)
Sleep restricted/hot	−3.23 ***	37.92 ***	31.15 ***	0.43 ***
	(0.34)	(5.74)	(4.90)	(0.09)
Normal sleep/cool	−0.97 ***	8.34 *	7.90 *	0.17 **
	(0.23)	(3.85)	(3.28)	(0.06)
Normal sleep/hot	−2.17 ***	26.35 ***	21.98 ***	0.36 ***
	(0.27)	(4.63)	(3.95)	(0.07)
R^2^	0.70	0.52	0.49	0.44
Adj. R^2^	0.69	0.49	0.47	0.41
Num. obs.	88	88	88	88
*F*(4,84)	49.3 ***	22.36 ***	20.31 ***	16.25 ***

*** *p* < 0.001, ** *p* < 0.01, * *p* < 0.05*.*

### 3.3. Subjective versus Objective Measures

A final analysis was conducted in which we compared the subjective measures in terms of correspondence with objective PVT Mean Reaction Time scores. The premise of the analysis was that an effective subjective measure should be more strongly associated with the preferred objective measure of fatigue. Linear mixed effects models were applied, with a random intercept for between-subjects variation, and fixed effects for the subjective measures. Data from the baseline, test, and recovery sessions were included in the analysis. Table 3 (Models 1–4) summarizes the results of a stepwise backward variable selection process, in which the worst performing indicator was progressively eliminated from an original set including Samn-Perelli Fatigue Scale, Alertness, Motivation and Pre-Performance. Models 4–7 allow comparison of each of the subjective indicators in isolation. Samn-Perelli Fatigue Scale was selected by the stepwise regression procedure as the best single predictor of PVT Mean Reaction Time scores. Samn-Perelli Fatigue Scale individually predicted 11.5% of the variance in PVT Mean Reaction Time scores. Although this effect size is not particularly high, it is nevertheless substantially higher than other subjective measures tested. Of the subjective measures, Samn-Perelli Fatigue Scale was most responsive to the experimental manipulations, and most highly correlated with the corresponding objective measure. We therefore concluded that Samn-Perelli Fatigue Scale was the most effective subjective measure of fatigue.

**Table 3:** Correspondence between measures of subjective fatigue and PVT.

Predictor	Model 1	Model 2	Model 3	Model 4	Model 5	Model 6	Model 7
(Intercept)	−0.009	−0.010	−0.000	0.004	0.033	0.033	0.061
	(0.083)	(0.082)	(0.083)	(0.083)	(0.088)	(0.085)	(0.088)
Samn-Perelli Fatigue Scale	−0.240 ***	−0.272 ***	−0.234 ***	−0.286 ***			
	(0.062)	(0.055)	(0.049)	(0.041)			
Alertness	0.092				0.270 ***		
	(0.082)				(0.044)		
Motivation	0.123	0.149 *	0.107			0.256 ***	
	(0.067)	(0.063)	(0.056)			(0.048)	
Pre-Performance	−0.132	−0.100					0.195 ***
	(0.074)	(0.067)					(0.048)
AIC	624.2	620.3	617	614	626	632	643
Var(Betw.)	0.481	0.469	0.488	0.496	0.567	0.516	0.566
Var(Resid.)_	0.317	0.321	0.318	0.320	0.324	0.347	0.356

*** *p* < 0.001, ** *p* < 0.01, * *p* < 0.05. **^** Table values are standardized beta-weights.

## 4. Discussion

Under conditions designed to increase fatigue, and in the presence of physical work tasks, the PVT was the most sensitive assay of fatigue in firefighters working a simulated deployment. The Samn-Perelli fatigue scale was the most sensitive of the subjective measures, and was the most highly correlated with PVT performance. The findings have both theoretical and practical implications.

The majority of laboratory studies examining changes in cognitive performance under conditions of sleep restriction minimize or eliminate physical activity. The current analysis however, demonstrates changes in PVT performance even in the presence of intermittent physical activity. This suggests that while some masking may be occurring [11], physical activity does not eliminate performance decrements associated with fatigue. In addition, the PVT was the most sensitive objective test of fatigue in this simulation, reinforcing the idea that simple and monotonous tasks such as vigilance are most susceptible to fatigue [26,27]. It is likely that the 10-min version of the PVT would show even greater sensitivity to fatigue. In addition, the PVT is a monotonous task requiring sustained attention. The level of fatigue elicited by the protocol may not have been sufficient to impact higher order tasks in the same manner. Lastly, the Samn-Perelli fatigue scale mirrored PVT performance more accurately than the visual analogue scales measuring alertness, motivation and performance prediction.

In terms of practical implications, one of the challenges in reducing fatigue-related accidents in occupational settings is to identify instances of elevated risk [3,4]. This requires assessment tools that reliably signal elevated fatigue before performance is impaired to the point of error. Objective and subjective tests that are sensitive to fatigue in all conditions, including in the presence of physical activity and other external stressors, may be applied as part of a fatigue risk management system. The ease with which the Samn-Perelli scale can be administered in the field, compared to objective performance tests, suggests it as the preferable option in such a system. However, the design and analysis of the current study allowed us to control for individual differences, something that is not possible in occupational settings. An alternative method is to use subjective scales in a repeated manner to determine changes within an individual, and/or in combination with other measures of performance.

It is important to note that the current work involved only day shifts and a moderate level of sleep restriction. Under more extreme conditions, including night work, the relationship may not hold. It is also relevant to note that: (1) The fatigue scale, although the most reliable subjective measure, only accounted for a small percentage of the variance; and (2) Our analysis did include several other subjective assessments of performance, none of which correlated well with PVT performance. This suggests that the specific questions asked of individuals in the field are important and should focus on tangible states such as those listed in the Samn-Perelli scale. While other subjective scales could be applied in both experimental and operational settings, the choice of the Samn-Perelli scale in this study was made on the basis of the specific descriptors. Fatigue as a concept is increasingly familiar to personnel in occupations that involve extended shifts or night/shift work. While debate continues as to the use of terms that describe sleepiness, fatigue, tiredness and alertness, the use of a fatigue scale to assess fatigue with the goal of developing more robust fatigue risk management scales was the logical choice.

While the aim of this study was to identify the most sensitive measures of fatigue in the presence of physical work, the generalizability of the findings to firefighter performance is an obvious question. The performance of the physical tasks during the simulation was not impacted by sleep [17], but reaction time and vigilance are both important faculties for preserving safety on the fireground. Therefore, the finding that PVT performance declines under certain conditions has practical implications, and indicates that at a moderate level of fatigue, lower order elements of cognitive performance are impacted. A next step in the research would involve monitoring of firefighting performance in live-fire scenarios.

## 5. Conclusions

The study found that neurobehavioral performance and subjective rating were impacted as expected under conditions likely to elicit elevated levels of fatigue, in spite of the repeated bouts of physical activity. This finding is important as the majority of studies examining performance changes in response to fatiguing conditions are conducted in the laboratory in the absence of physical activity, whereas high-risk occupations do involve physical work (e.g., emergency services, mining, military *etc.*). Importantly, the Samn-Perelli fatigue scale most strongly correlated with changes in cognitive performance, suggesting it may be a useful self-assessment tool in certain occupational settings. These findings need to be tested further in live-fire scenarios and also during night shifts.
